# From Realism to Learner Engagement: Rethinking Fidelity in Simulation-Based Education

**DOI:** 10.2196/84684

**Published:** 2026-02-23

**Authors:** Julien Pico, Jean-Noel Evain, Christina Aron, Gilles Martin, Ilian Cruz-Panesso, Leonida-Mihai Georgescu, Issam Tanoubi

**Affiliations:** 1 Centre Hospitalier Universitaire de Montpellier, Montpellier, Occitanie, France; 2 Centre d'apprentissage des attitudes et habiletés cliniques (CAAHC), Université de Montréal, Pavillon Roger-Gaudry, 2900, boul. Édouard-Montpetit, 8e étage, Montréal, QC, H3T 1J4, Canada, 1 5142493006; 3 Direction de l'enseignement et de l'Académie CHUM (DEAC), Montréal, QC, Canada; 4 Centre de Recherche en Pédagogie de la Santé (CRPS), Faculté de Médecine, Université de Montréal, Montréal, QC, Canada

**Keywords:** fidelity, fiction contract, learning, simulation training, medical education

## Abstract

Simulation has become an essential pedagogical tool in health professions education, traditionally valued for its ability to approximate clinical practice. Higher simulation fidelity is often assumed to directly enhance learner engagement and improve educational outcomes; however, this assumption oversimplifies a complex relationship. Fidelity is multidimensional, encompassing physical, emotional, and contextual dimensions, as well as qualitative and quantitative considerations, each influencing learners’ perception of realism in distinct ways. Engagement is shaped not only by these dimensions of fidelity but also by intrinsic factors such as motivation, prior experience, stress, and emotional resilience, and by extrinsic factors including instructional design, facilitation, debriefing, and psychological safety. A central mediator in this process is the fiction contract, an implicit agreement that enables learners to suspend disbelief and engage authentically despite inherent limitations in realism. Technological sophistication alone does not necessarily translate into greater educational impact. Rather, fidelity should be intentionally aligned with learning objectives: advanced patient simulators may support procedural training, standardized patients may enhance communication skills, and task trainers may optimize focused psychomotor practice. This viewpoint advocates for a goal-oriented, multimodal approach that redefines high-fidelity simulation not as the pursuit of maximum realism, but as the deliberate alignment of fidelity with pedagogy to optimize learner engagement and educational effectiveness.

Simulation is a realistic representation of clinical practice in health care education, guiding learners to the apex of Miller’s pyramid of competence[1]. In the context of health professions education, simulation plays a pivotal role in developing practical competence in clinical environments, specifically the acquisition of skills in real conditions and practical knowledge. The primary educational goal is to engage learners in Kolb’s experiential learning cycle [2]. However, this goal often assumes that student engagement with simulation is directly and linearly linked to its fidelity [3]. But how valid is this assumption, and along which dimensions [4]? Simulation-based education in health professions has long been influenced by the assumption that higher fidelity directly enhances learning. However, foundational studies have questioned this linear relationship. Norman et al [5] demonstrated that the level of simulation fidelity had minimal impact on the transfer of learning, emphasizing instead the role of instructional design. Building on this, Hamstra et al [6] introduced the idea of functional task alignment, suggesting that fidelity should be defined relative to educational goals rather than technical sophistication. Petrosoniak et al [7] further argued that simulation design must serve both learner needs and broader health care system objectives and Dieckmann et al [8] emphasized the complex and socially constructed nature of realism in simulation-based education. These perspectives provide the theoretical backdrop for our reevaluation of fidelity and its relationship to learner engagement. This position, while increasingly supported in the literature, remains debated and calls for a more nuanced, evidence-informed understanding of when and how fidelity contributes to learning. In this essay, we revisit the concept of fidelity in simulation, analyze its multiple components, and evaluate their potential impact on student engagement. We will then suggest a redefinition of high-fidelity simulation aligned with educational objectives and highlight the significance of the fiction contract as a precondition for any simulation activity.

## Qualitative and Quantitative Fidelity

Simulation fidelity encompasses two key dimensions—qualitative and quantitative—which are used here as a heuristic lens rather than as a comprehensive or exclusive framework [9,10]. Other frameworks describe fidelity and ecological validity across broader sociotechnical dimensions, including tasks, technologies, environments, systems, and professional. Qualitative fidelity refers to the degree to which simulated elements authentically mirror real-world counterparts. For instance, incorporating equipment used in clinical practice, such as an actual anesthesia machine, into an operating room simulation enhances this qualitative dimension. In contrast, quantitative fidelity pertains to the number and richness of elements included in the simulation activity compared to real life. For example, a patient’s fall in a hospital room with a suitcase full of clothes, an overturned table, and shattered glass on the floor increases quantitative fidelity. However, increasing the number of elements within a scenario does not inherently enhance educational fidelity and may introduce unnecessary cognitive load if not aligned with learning objectives.

A higher level of fidelity allows learners to become immediately immersed in a real-world context, reducing the need for imagination and fostering stronger situational awareness. Conversely, lower fidelity requires learners to engage their imagination continuously, which can disrupt natural responses and divert cognitive resources away from patient care. This additional cognitive load, resulting from efforts to compensate for missing realism, may ultimately impair performance and reduce learning effectiveness [11,12]. These dimensions of fidelity further interact with qualitative and quantitative considerations and must be interpreted relative to the intended educational goals.

## The Dimensions of Fidelity in Simulation-Based Medical Education

Three major dimensions of fidelity are frequently described in the literature. (1) Physical fidelity refers to the degree of visual and functional resemblance between simulated elements and their real-world counterparts. (2) Emotional fidelity pertains to elements, often sensory or contextual, that evoke emotional responses in participants. (3) Contextual fidelity relates to the progression of a situation as it would unfold in real clinical settings. Each of these types of fidelity influences the learner’s perception of realism [13]. While the impact of physical resemblance may seem obvious, the effects of the other forms of fidelity are less straightforward, as some studies suggest that simulation can elicit emotional and physiological responses comparable to real clinical situations despite limitations in physical realism [14].

Emotional fidelity enhances realism by eliciting the same emotions in the learner that would arise in a real situation [15]. For example, an actor portraying a distraught and tearful father during the resuscitation of his newborn could significantly heighten the realism of the simulation activity. These emotional responses are crucial for several reasons. First, they may create cognitive load similar to real-life stress, forcing learners to manage their emotions, regulate their reactions, and remain focused on patient care. Without this exposure in simulation, learners may face such emotions for the first time in real clinical situations, potentially resulting in suboptimal performance [16]. Emotionally charged situations also promote memory retention [17], a desirable outcome in education. However, this type of fidelity could have adverse effects if the learner has previously experienced a similar emotional situation or if there is emotional overload, potentially leading to “freezing” or memory suppression [18].

Contextual fidelity is equally critical from an educational perspective. The timing of the simulation activity progression must respect the real-life temporal context. Accelerating or slowing down events during the activity, such as receiving the results of a diagnostic test in 2 minutes when it should take 20 or allowing the patient’s condition to deteriorate slowly to give participants time to find the “solution,” could detract from experiential learning. The decisions made by the learner are tailored to the designed activity, which might not be applicable to a real clinical setting or might even be wrong. Contextual fidelity is even more critical in the context of in situ simulation. It is essential for uncovering latent safety threats within the clinical environment [19].

## Relationship Between Simulation Fidelity and Learner Engagement in the Simulation Activity

It is commonly assumed that greater fidelity leads to stronger learner engagement, as participants perceive the activity as more realistic and therefore more pedagogically useful [20]. However, this assumption is not consistently supported, as studies comparing simulators of differing fidelity have shown similar learner performance and decision-making outcomes, even when using less technologically advanced simulation modalities [21,22]. Other internal factors may modulate this engagement, such as the learner’s motivation, performance-related stress, and evaluation anxiety; the emotional intelligence of the instructor and the debriefer; and the clinical complexity of the simulation activity.

**Figure 1.**
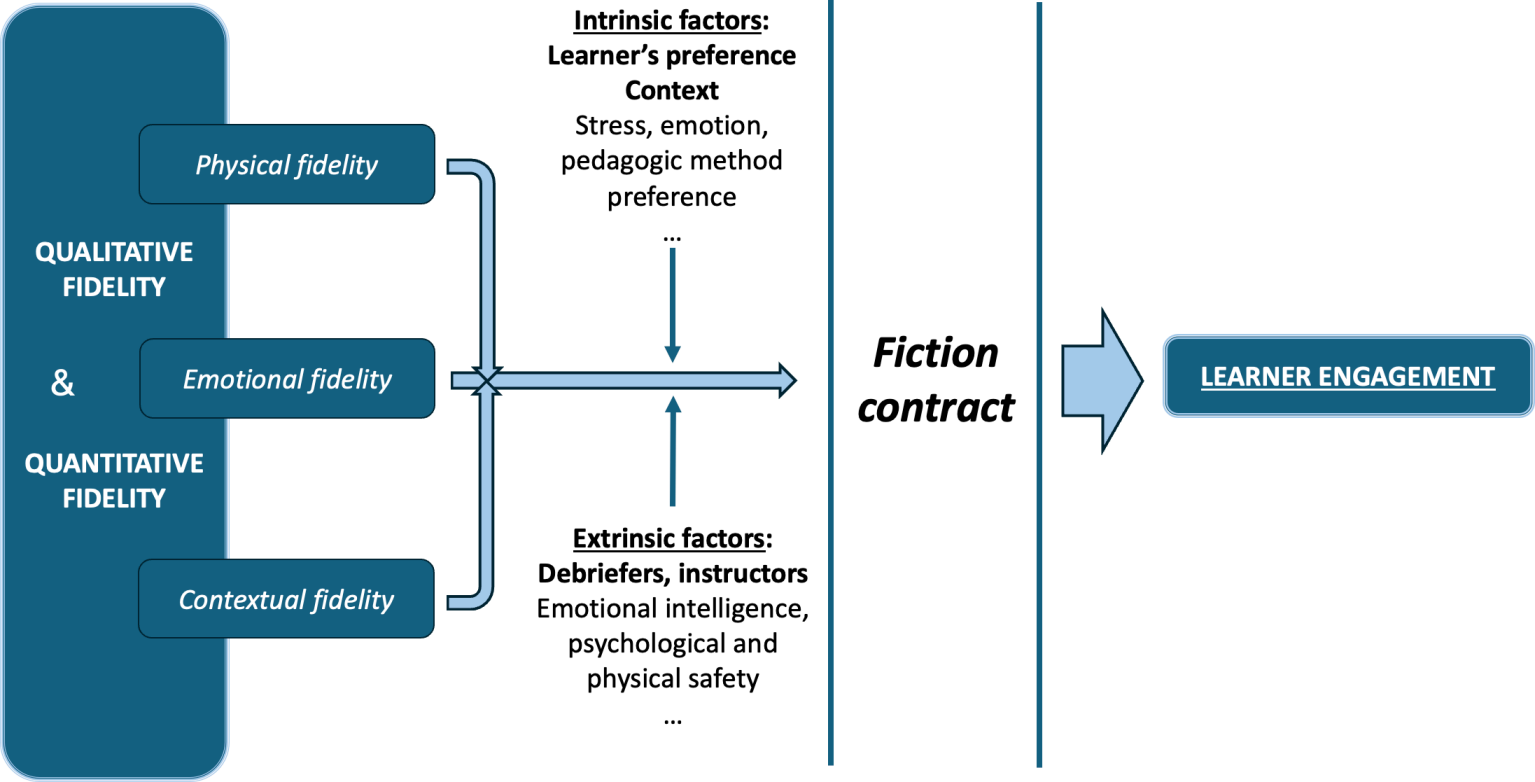
Modulating factors of learner engagement during medical simulation.

In this context, the importance of the *fiction contract* becomes paramount [3,23,24]. The fiction contract is an implicit, verbally acknowledged agreement between the instructor and the learners. The fiction contract acts as a mediator between fidelity and learner engagement by enabling learners to suspend disbelief and engage authentically despite inevitable limitations in simulation realism. During the prebriefing, the instructor is encouraged to clarify the limitations of the simulation, describe the team’s efforts to enhance fidelity, and explain that the realism of the situation also stems from the authenticity of the learners’ responses. By explicitly framing expectations and acknowledging these constraints, the fiction contract allows learners to focus on the learning task rather than on imperfections in realism. This approach encourages participants to “buy into” the activity, treating the simulated situation as if it were real. The ultimate goal is to leverage simulation as a pedagogical tool at the “show how” stage of Miller’s pyramid, thereby fostering engagement in the experiential learning cycle through simulated practice (Figure 1).

## Goal-Oriented Multimodal Simulation Fidelity

Our proposal to redefine high-fidelity simulation as the alignment of simulation features with educational objectives is consistent with existing literature that challenges the traditional focus on realism. Norman et al [5] and Hamstra et al [6] both caution against overemphasizing fidelity, highlighting instead the importance of instructional relevance and cognitive congruence. Petrosoniak et al [7] emphasize a systems-level view, advocating for simulation design that is responsive to functional demands rather than esthetic realism. These frameworks collectively support our goal-oriented approach, reinforcing the idea that the effectiveness of a simulation lies not in its resemblance to reality, but in how well it facilitates the intended learning outcomes [25,26]. Simulation modalities must be intentionally selected based on their alignment with specific learning goals rather than technological sophistication alone [27,28].

The inherent limitations of fidelity and the near impossibility of making a simulation fully resemble real life compel instructors to align simulation design with educational objectives. For example, in a simulation focused on a critical clinical situation requiring urgent intervention, the emphasis should be on the progression of the patient’s clinical deterioration, the simulated patient’s vital signs, and the dynamic clinical response to treatment. Conversely, in a simulation aimed at managing the emotional response of a patient or family members discovering the death of a loved one should prioritize the performance of the actors portraying the simulated family [29]. In the first case, the use of an advanced patient simulator is essential, whereas actors are more appropriate in the second activity. In the same vein, a task trainer will be most useful when acquiring the skill of vaginal examination [30]. Therefore, fidelity should be tailored to the learning goal, rather than relying on the use of an advanced patient simulator. A highly technological simulation is not necessarily synonymous with high fidelity and may, in fact, lead to resource overconsumption [31] with limited educational benefit. To maximize impact, simulation educators must keep learning objectives at the center, carefully considering the most effective simulation method—be it actors, advanced patient simulators, or task trainers—and aligning the fidelity to meet that objective [32-34].

Our argument, that higher fidelity is not inherently superior and may even be counterproductive when misaligned with learning goals, is consistent with a growing body of literature. Studies have demonstrated that the educational impact of simulation does not correlate directly with its realism, but rather with how well the simulation supports cognitive and procedural learning; these studies advocate for simulation design that serves specific system and learner needs rather than aiming for maximal fidelity [5-7].

However, we acknowledge that this perspective is not without debate. Some educators argue that high-fidelity simulations can enhance psychological immersion or better prepare learners for complex clinical environments. While this may be true in specific contexts, such as team-based crisis management or emotional preparedness, we suggest that these benefits arise not from fidelity per se, but from how the design elements align with targeted competencies. In fact, pursuing high levels of realism without clear pedagogical justification can lead to resource inefficiencies or even cognitive overload, detracting from the intended learning outcomes.

Rather than taking a position against fidelity, our goal is to promote intentionality in its use. By considering fidelity as a flexible, goal-oriented construct rather than a fixed technological standard, educators can make more informed decisions that balance effectiveness, efficiency, and learner experience.

## Conclusion

Simulation fidelity is multidimensional, encompassing physical, emotional, and contextual dimensions that shape learners’ perception of realism. However, fidelity alone does not ensure effective learning. Rather than maximizing realism, educators should redefine high-fidelity simulation as the deliberate alignment of fidelity with pedagogy, treating fidelity not as a fixed standard but as a flexible design tool that serves pedagogical intent above all.
